# Lipid-Based Nanosystems for the Topical Application of Ferulic Acid: A Comparative Study

**DOI:** 10.3390/pharmaceutics15071940

**Published:** 2023-07-12

**Authors:** Maddalena Sguizzato, Francesca Ferrara, Markus Drechsler, Anna Baldisserotto, Leda Montesi, Stefano Manfredini, Giuseppe Valacchi, Rita Cortesi

**Affiliations:** 1 Department of Chemical, Pharmaceutical and Agricultural Sciences (DoCPAS), University of Ferrara, I-44121 Ferrara, Italy; sgzmdl@unife.it (M.S.); frrfnc3@unife.it (F.F.); 2 Bavarian Polymer Institute (BPI) Keylab “Electron and Optical Microscopy”, University of Bayreuth, D-95440 Bayreuth, Germany; markus.drechsler@uni-bayreuth.de; 3 Department of Life Sciences and Biotechnology, University of Ferrara, I-44121 Ferrara, Italy; bldnna@unife.it (A.B.); leda.montesi@unife.it (L.M.); smanfred@unife.it (S.M.); 4 Department of Environmental and Prevention Sciences, University of Ferrara, I-44121 Ferrara, Italy; gvalacc@ncsu.edu; 5 Plants for Human Health Institute, Department of Animal Science, NC Research Campus Kannapolis, NC State University, Kannapolis, NC 28081, USA; 6 Department of Food and Nutrition, Kyung Hee University, Seoul 130-701, Republic of Korea; 7 Biotechnology Interuniversity Consortium (C.I.B.), Ferrara Section, University of Ferrara, I-44121 Ferrara, Italy

**Keywords:** ferulic acid, antioxidant molecules, transferosomes, vesicles, monoolein aqueous dispersions, topical application, cryo-TEM

## Abstract

In this study, we examined and compared two different lipid-based nanosystems (LBNs), namely Transferosomes (TFs) and Monoolein Aqueous Dispersions (MADs), as delivery systems for the topical application of Ferulic Acid (FA), an antioxidant molecule derived from natural sources. Our results, as demonstrated through Franz-cell experiments, indicate that the LBNs produced with poloxamer 188 in their composition create a multilamellar system. This system effectively controls the release of the drug. Nonetheless, we found that the type of non-ionic surfactant can impact the drug release rate. Regarding FA diffusion from the MAD, this showed a lower diffusion rate compared with the TF. In terms of an in vivo application, patch tests revealed that all LBN formulations tested were safe when applied under occlusive conditions for 48 h. Additionally, human skin biopsies were used to determine whether FA-containing formulations could influence skin tissue morphology or provide protection against O_3_ exposure. Analyses suggest that treatment with TFs composed of poloxamer 188 and MAD formulations might protect against structural skin damage (as observed in hematoxylin/eosin staining) and the development of an oxidative environment (as indicated by 4-hyroxinonenal (4HNE) expression levels) induced by O_3_ exposure. In contrast, formulations without the active ingredient did not offer protection against the detrimental effects of O_3_ exposure.Inizio modulo.

## 1. Introduction

In the last few decades, lipid-based nanosystems (LBNs) have attracted significant interest as matrices for the controlled release of active molecules aimed at simultaneously improving their bioavailability and to reduce side effects [1]. In the present study, two different LBNs, namely transferosomes (TFs) and Monoolein Aqueous Dispersions (MADs) have been considered and compared as delivery systems for the topical application of ferulic acid (FA), a natural antioxidant molecule widely used in skincare products due to its beneficial effects on skin structure and the vascular endothelium [2,3].

Transferosomes can be defined as liposomes consisting of phosphatidylcholine and an edge activator [4]. The composition of these vesicles consists of hydrophobic and hydrophilic moieties able to lodge active molecules characterized by different solubilities. In addition, TFs are able to navigate themselves through narrow passages, allowing intact vesicles to penetrate tissues [5,6,7].

The self-assembly in water of amphiphilic lipids such as monoglycerides gives rise to the formation of many lyotropic liquid crystalline nanostructures such as micelles, lamellae, and hexagonal and cubic phases, depending on the temperature and water content of the system [1,8,9,10,11,12]. These structures are known as MADs. Regardless of the production mechanism, the obtained MAD relies on stabilizing agents for preservation. The most commonly used surfactants are polymers belonging to the poloxamer family, but molecules such as methoxy polyethylene glycol (mPEG) can also be employed.

In this study, our goal was to identify a delivery system capable of enhancing the performance of the drug. To that end, we designed, produced, and characterized several LBNs based on size, morphology, and stability. We also conducted in vitro, ex vivo, and in vivo studies to evaluate the effectiveness of the selected formulations for the topical application of natural molecules possessing antioxidant properties.

In particular, the protective effect of FA provided by the different formulations against detrimental effect on the cutis is evaluated in human skin biopsies exposed to ozone (O_3_) since this air pollutant is able to promote the onset of a variety of cutaneous pathological conditions. Indeed, O_3_ is one of the most toxic environmental agents known due to its ability to trigger inflammatory and oxidative stress reactions within the human skin (OxInflammation) [13]. 

## 2. Materials and Methods

### 2.1. Materials

Glyceryl monooleate RYLO MG 19 was supplied by Danisco Cultor (Grindsted, Denmark). Phosphatidylcholine (PC) Phospholipon 90G was purchased from Lipoid GmbH (Ludwigshafen, Germany). Ferulic acid (trans-Ferulic acid, trans-4-Hydroxy-3-methoxycinnamic acid, FA, purity ≥ 99.0%), cholesterol (CH), Tween 20 (T), Span 20 (S), the copolymer poly(ethylene glycol)-block-poly(propylene glycol)-block-poly(ethylene glycol) poloxamer 188 (P), and all other materials and solvents were obtained from Merck KGaA (Darmstadt, Germany). DMEM High Glucose Media, fetal bovine serum (FBS), Penicillin, Streptomycin, Amphotericin B, sodium citrate buffer, and 10% neutral-buffered formalin (NBF) were purchased from Gibco, ThermoFisher Scientific (Waltham, MA, USA). The 4-Hydroxy-nonenal (4HNE) primary antibody was supplied by Merck Millipore (Burlington, MA, USA), whereas the fluorochrome-conjugated secondary antibody DAPI and Fluoromount-G™ Mounting Medium were obtained from Invitrogen, ThermoFisher Scientific (Waltham, MA, USA).

### 2.2. Lipid-Based Nanosystem Preparation 

TFs were prepared by adopting the thin-layer hydration method [14]. Specifically, a mixture of PC, CH, and surfactant (4:2:1 mol/mol/mol) and an FA solution were subjected to evaporation in an organic solvent mixture (chloride/methanol, 1:1 by volume) using a rotary evaporator (Rotavapor R-200, Büchi Italia, Cornaredo, Italy) until a lipid film was deposited on the glass wall of the round flask. Afterwards, the obtained film was hydrated alternately with Palitzsch buffer (B) [15] or Poloxamer 188 (P) solution (2.5% *w*/*w*) until a final concentration of 25 mg/mL in terms of total lipids and 2 mg/mL of FA content was reached. The hydrated film was then swirled and sonicated in a sonic bath at 25 °C for 30 min. 

MADs were prepared by emulsifying monoolein and poloxamer 188 in water followed by hot homogenization at 60 °C and 15,000 rpm for 5 min (Ultra Turrax, Janke & Kunkel, Ika-Werk, Sardo, Italy). The preparation was then cooled at room temperature in glass vials as previously described [16]. FA-containing MADs were prepared by adding 0.2% by weight of the total solid mixture of FA into the molten monoolein/emulsifier mixture. After FA dissolution, the aqueous phase was added, and the dispersion was prepared as described above.

Table 1 reports the composition of the prepared LBNs.

### 2.3. Lipid-Based Nanosystem Characterization 

The size of the obtained lipid-based nanosystems was assessed at 25 °C and a 90° angle by means of a Zetasizer Nano S90 (Malvern Instr., Malvern, UK) mounted with a 5 mW helium–neon laser with a 633 nm output wavelength. The run time was around 180 s, and obtained data were interpreted by applying the “CONTIN” method [17].

The morphology of lipid-based nanosystems was visualized using a Zeiss EM922Omega Cryogenic Transmission Electron Microscope (Cryo-TEM) at a temperature below −175 °C. Vitrified samples were transferred to a TEM using a cryoholder (CT3500, Gatan Inc., Pleasanton, CA, USA) [18]. Afterwards, specimens were examined with doses of about 1000–2000 e/nm^2^ at 200 kV, and images digitally recorded (CCD camera UltraScan 1000, Gatan Inc., Pleasanton, CA, USA) were processed using the GMS 1.4 software (Gatan Inc., Pleasanton, CA, USA). 

### 2.4. Encapsulation Efficiency of FA in Lipid-Based Nanosystems

The content of FA within the produced lipid-based nanosystems was expressed by the encapsulation efficiency (EE). Specifically, for transferosomes, 300 µL of each formulation (Microcon YM-10 membrane NMWCO 10 kDa, Sigma-Aldrich, St. Louis, MO, USA) was subjected to 20 min ultracentrifugation at 8000 rpm (Spectrafuge™ 24D Digital Microcentrifuge, Woodbridge, NJ, USA). Subsequently, methanol (1:10, *v*/*v*) was added to 100 µL of the retentate and the drug quantified by UV to obtain the EE by applying Equation (1).

EE = FA/FA_T_ × 100 (1)

in which FA is the amount of ferulic acid as determined by UV analyses, while FA_T_ is the total weighed drug used to prepare the formulation. A double-ray UV/Vis spectrometer (Lambda19 UV/VIS/NIR Spectrometer, Perkin Elmer, Waltham, MA, USA) operating at 318 nm in 1 mL quartz cuvettes was employed to quantify the drug using an FA extinction coefficient (E^mM^) of 18.6 mM^−1^cm^−1^.

In the case of MADs, 100 μL of a dispersion was diluted with 900 μL of methanol and stirred for 3 h. Afterwards, the sample was analyzed by UV as described above.

### 2.5. Prediction of Long-Term Stability

The chemical stability of FA in TFs and MADs was evaluated by UV for one month after production and shelf-life values were calculated as reported below [19].

The concentration over time of FA within LBNs, as determined by UV as mentioned above (Section 2.4.), has been considered for the prediction of long-term stability of formulations [20]. In particular, Log(FA residual content, %) was plotted against time, and the slopes (m) were calculated by linear regression. The obtained m value is then used in Equation (2) for the determination of k values [21,22].

k = m × 2.303 (2)

Shelf-life values (e.g., the time for 10% loss, t_90_) and half-life (e.g., the time for 50% loss, t_1/2_) were then calculated by applying Equations (3) and (4).

t_90_ = 0.105/k (3)

t_1/2_ = 0.693/k (4)

### 2.6. In Vitro Diffusion Experiments

A battery of four Franz cells (1 cm diameter orifice, 0.78 cm^2^ exposed diffusion area, LGA, Berkeley, CA, USA) associated with the mixed cellulose–ester membrane was employed to determine FA diffusion. Before mounting the cells, the membranes were hydrated in distilled water at room temperature for 1 h. PBS (pH 7.4) was adopted as the recipient phase to mimic physiological conditions [23]. A volume of 5 mL of PBS, kept at 32 ± 1 °C, was poured into the receptor compartment and magnetically stirred at 300 rpm over the whole experiment [24]. Each donor compartment was loaded with 1 mL of each formulation in deep contact with the surface membrane. During the 8 h experiment, 300 µL of the receptor phase was withdrawn and subjected to UV analysis. Meanwhile, the removed volume was replaced with fresh PBS. The mean values ± standard deviations of diffused FA, expressed as µg/cm^2^, were plotted as a function of time [25]. Diffusion coefficients (Js) were extrapolated from the slope of the linear portion of the accumulation curve and expressed as normalized fluxes (Jn) by dividing by the FA concentration in the analyzed form, expressed in mg/mL. The release-kinetics parameters were evaluated by mathematical models [26], and R^2^ values were taken as a reference to evaluate the FA-release mechanism [27].

### 2.7. In Vitro Antioxidant Capacity

LBN antioxidant capacities were evaluated with DPPH and FRAP assays [28].

The DPPH assay makes it possible to estimate the hydrogen-donating capacity of an antioxidant substance to convert the stable free-radical DPPH into 1,1-diphenyl-2-picrylhydrazyl. This radical reaction is accompanied by an intense violet to light yellow colorimetric reaction, which can be measured by considering the percentage of absorbance reduction at 517 nm using a UV–Vis spectrophotometer (UV/VIS ONDA Touch UV-31 Scan Spectrophotometer, Sinergica Soluzioni S.r.l., Milan, Italy) as previously described [29]. The percentage of the radical scavenging capacity was obtained by applying Equation (5).

DPPH radical scavenging capacity (%) = [1 − (A1 − A2)/A0] × 100 (5)

where A0 is the absorbance of the control (without FA), A1 is the absorbance in the presence of FA, and A2 is the sample absorbance in the absence of the DPPH reagent. 

Aliquots of 0.750 mL of FA (as solutions or LBNs) at different concentrations were added to 1.5 mL of DPPH as a methanol solution. The IC_50_ values are determined by means of the previously obtained calibration curve. The values (μg/mL) are the mean of three independent experiments ± standard deviation.

The FRAP assay is used to quantify the ability of a sample to reduce ferric ions (Fe^3+^) to ferrous ions (Fe^2+^) under acidic conditions in the presence of 2,4,6-tripyridyl-s-triazine (TPTZ) [30]. The presence of FA led to the reduction of the Fe^3+^–TPTZ complex to the ferrous form, resulting in the formation of a blue color with a maximum absorption wavelength at 593 nm. Using Trolox as a reference, the FRAP antioxidant activity is indicated as μmol Trolox equivalent/g of FA.

### 2.8. In Vivo Patch Test

An in vivo irritation test was carried out to evaluate the effect of an LBN applied in a single dose on intact human skin. The occlusive patch test was carried out on 20 healthy volunteers of both sexes following the study N° 170583 describing the protocol criteria for skin compatibility testing of potentially cutaneous irritant cosmetic ingredients on human volunteers [31,32,33]. Study approved by the Ethics Committee of the University of Ferrara, Italy. The volunteers (a) affected by dermatitis, (b) sensible to allergic skin reactions, or (c) undergoing treatment with anti-inflammatory drugs (either steroidal or non-steroidal) were excluded from the study. The remaining 20 gave their written consent to the experimentation. An aluminum Finn chamber (Bracco, Milan, Italy) carrying 10 mg of the LBN formulation was applied onto the forearm or the back skin and safeguarded with self-sticking tape. The studied formulation was loaded onto the Finn chamber using an insulin syringe and left in contact with the skin surface for 48 h. After patch removing (15 min and 24 h post-application) and skin cleaning, the presence of erythema and/or edema reactions was evaluated. Erythematous reactions (sorted as light, clearly visible, and moderate/serious erythema degree) and other irritation reactions were expressed as a percentage of the total number of reactions occurring in the volunteers. 

### 2.9. Ex Vivo Studies

#### 2.9.1. Culture of Human Skin Biopsies and Treatment

Skin punch biopsies (12 mm) were collected from elective abdominoplasties purchased from HKB Surgery Hospital in Huntersville, NC. After trimming the subcutaneous fat from the skin, the 12 mm biopsies were washed in PBS and placed in 6-well plates containing 1 mL of fresh DMEM High Glucose Media containing 10% FBS, 100 U/mL Penicillin, 100 mg/mL Streptomycin, and 0.25 mg/mL Amphotericin B (Gibco, ThermoFisher Scientific, Waltham, MA, USA). Skin explants were left overnight in an incubator at 37 °C and 5% CO_2_ to recover; these were then treated with 20 µL of the ethanol solution (Sol) and different LBNs that were unloaded (Sol, TB1, TP1, MAD) or FA-loaded (Sol-FA, TB1-FA, TP1-FA, MAD-FA) by applying them onto the skin using a glass rod. Twenty-four hours after the treatment, skin biopsies were exposed to 0.4 ppm of ozone (O_3_) for 4 h by using an ozone generator, as previously described [34]. Samples were collected 6 and 24 h after the end of the O_3_ exposure and processed for the subsequent analysis. The experiment was conducted in three different donors.

#### 2.9.2. Hematoxylin/Eosin Staining

Human skin biopsies were fixed in 10% neutral-buffered formalin (NBF) for 48 h at 4 °C and then dehydrated in a series of alcohol gradients using xylene. After paraffin embedding, 4 µm tissue sections were cut using a microtome and then subjected to hematoxylin/eosin staining [34]. The obtained slides were mounted on glass using a toluene-based solution and imaged using an EVOS Cell Imaging System (ThermoFisher Scientific, Waltham, MA, USA) equipped at 40x magnification.

#### 2.9.3. Immunohistochemistry

Immunohistochemistry was performed as elsewhere reported [35]. Briefly, 4 µm skin biopsy sections were deparaffinized in xylene, rehydrated in decreasing alcohol gradients, and then subjected to antigen demasking. Slides were immersed in a solution of 10 mM sodium citrate buffer (AP-9003-500, ThermoFisher Scientific, Waltham, MA, USA) (pH 6.0) and then heated in a water bath at a sub-boiling temperature of 95 °C for 10 min. Samples were cooled down to RT for 30 min, washed 2 times in PBS for 5 min and then blocked in a 2% BSA/PBS solution for 45 min at RT. Samples were then incubated overnight at 4 °C with the primary antibody 4-Hydroxy-nonenal (4HNE) (AB5605 Merck Millipore, Burlington, MA, USA) at a dilution of 1:400 in a solution of 0.25% BSA/PBS. The next day, the sections were washed in PBS 3 times for 5 min and then incubated for 1 h at RT with the fluorochrome-conjugated secondary antibody (A11008 Alexa Fluor 488; Invitrogen, ThermoFisher Scientific, Waltham, MA, USA) diluted 1:1000 in a solution of 0.25% BSA/PBS. After washing in PBS, nuclei were stained for 1 min with DAPI (D1306, Invitrogen, ThermoFisher Scientific, Waltham, MA, USA) diluted 1:50′000 in PBS. Slides were washed again in PBS and mounted onto glass using Fluoromount-G™ Mounting Medium (cat. 00-4958-02, ThermoFisher Scientific, Waltham, MA, USA). The Zeiss LSM10 microscope equipped at 40× magnification was used to image the fluorescence of 4HNE, and the signal was quantified using the ImageJ software.

### 2.10. Statistical Analysis

The statistical analysis was conducted by employing GraphPad Prism 9 software (Version 9.4.1. (458), GraphPad Software Inc., La Jolla, CA, USA). The variance between groups was measured by 2-way ANOVA, followed by Tukey’s post hoc test. A value of *p* < 0.05 was considered statistically significant. Data are expressed as mean ± SD of duplicate determinations from three independent experiments.

## 3. Results and Discussion

### 3.1. Production and Characterization of FA-Loaded Vesicular Systems

To overcome the problems related to low bioavailability after the administration of FA, a pre-formulation study was conducted [36]. In this view, LBNs with different peculiarities ascribable to their composition, such as TFs and MADs, have been designed with the aim of obtaining a suitable formulation for FA with good characteristics in terms of its encapsulation efficiency, controlled release, physicochemical stability, antioxidant effect, and skin application. TFs (see Table 1 for composition) were produced using the thin-layer hydration method. In particular, two hydration media, the Palitzsch buffer (B) and a 2.5% *w*/*w* solution of Poloxamer 188 (P) were employed, and Span 20 (1) and Tween 20 (2) were selected as edge activators. In contrast, MADs (see Table 1) were obtained by the emulsification method, as reported in Section 2.2.

**Figure 1:**
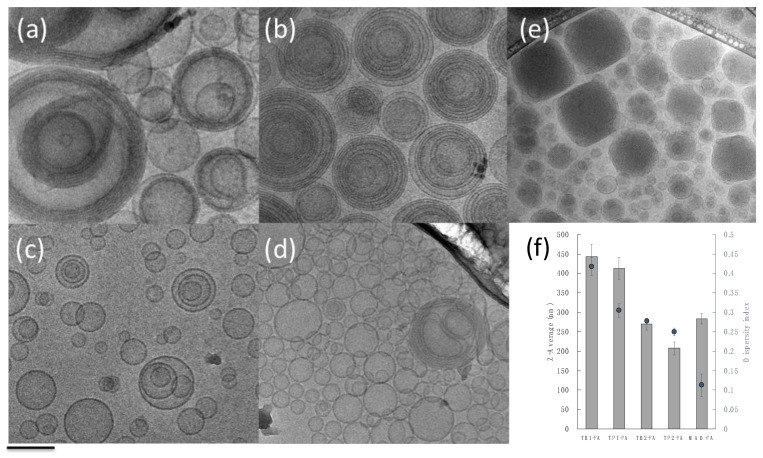
Cryogenic-TEM images of TB1-FA (**a**), TP1-FA (**b**), TB2-FA (**c**), TP2-FA (**d**), and MAD-FA (**e**), and a graphical representation of their dimensional parameters (**f**). Scale bar represents 200 nm.

After production, the FA-loaded LBNs were characterized in terms of their morphology, size, and size distribution (Figure 1 and Table 2). 

As depicted in Figure 1, the morphological analysis, performed by mean of cryogenic TEM, indicated that mono- or multilamellar TFs were obtained with few differences, depending on the edge activator and the hydration medium utilized. In particular, the use of Span 20 (TB1-FA and TP1-FA) gave rise to concentric multilamellar vesicles with sizes larger than 400 nm; in the case of TB1-FA, a population of monolamellar vesicles is present, while in the case of TP1-FA, monolamellar vesicles are almost absent. On the other hand, it should be noted that the presence of Tween 20 led to the formation of mainly monolamellar vesicles (TB2-FA and TP2-FA) of lower average size (Figure 1f).

In the case of MAD-FA, the presence of vesicles and cubic structures with a mean size distribution around 280 nm was evident [37].

Concerning the dimensional stability of the formulations, size and polydispersity were monitored by PCS over one month. The values reported in Table 2 revealed that both TFs and MADs preserved the dimensional distribution along the investigated time period. 

### 3.2. Encapsulation Efficiency and Stability Studies of FA

The encapsulation efficiency of FA in LBNs was evaluated after production and the stability of drug content in the produced LBN was monitored over time for up to 30 days after production. 

**Figure 2:**
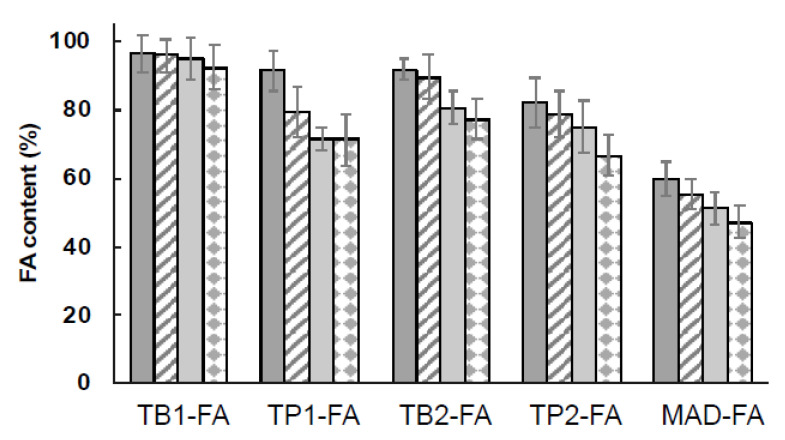
Encapsulation efficiency and percentage of the drug content of FA in the produced LBNs over time, namely 1 (dark grey), 7 (stripes), 15 (light grey), and 30 days (squares) after production. Data are the mean of four independent experiments ± s.d.

Figure 2 shows the FA content in TFs and MADs, expressed as a total percentage, as reported in Equation (1). The highest encapsulation of the drug was obtained with TF formulations, where the percentages of the FA content ranged between 83% and 97%, while in the case of MADs, the FA content after production was around 60%. In particular, for TFs, the encapsulation in TB1-FA decreased by only 5% after 30 days, while in the case of TP1, TB2, and TP2, the content of FA reached around 70%. Hence, TFs could be considered a promising system for FA loading. 

Concerning MAD-FA, the drug content tended to decrease over time. It could be supposed that the presence of heterogeneous structures in the formulation, such as cubosomes, particles, and vesicles, can affect the chemical stability of the molecule, leading to reduced entrapment efficiency. Indeed, the lipid nature of nanostructures composing MADs may interfere with the carrying of amphiphilic molecules such as FA with respect to sole vesicular nanosystems, in which the accommodation of the amphiphilic drug can occur on the lipid bilayer or in the aqueous core of the vesicles.

In order to better understand the ability of formulations to maintain FA encapsulation and to predict the stability of the loaded drug over time, shelf-life (t_90_) and half-life (t_1/2_) values were calculated for each formulation using Equations (3) and (4), and the results are reported in Table 3. Shelf life indicates the time at which the drug concentration was reduced by 10% (t_90_), while half-life indicates the time at which the drug concentration was reduced by 50% (t_1/2_). 

From the reported data, it is evident that TFs were able to maintain FA stability for the longest time, while MADs presented a short stability, in agreement with the low values of encapsulation efficiency discussed above. Remarkably, TB1-FA displayed t_1/2_ values 5-fold longer with respect to MADs, confirming that the vesicular system serves as a more appropriate vehicle for FA. 

### 3.3. In Vitro FA Diffusion Kinetics

**Figure 3:**
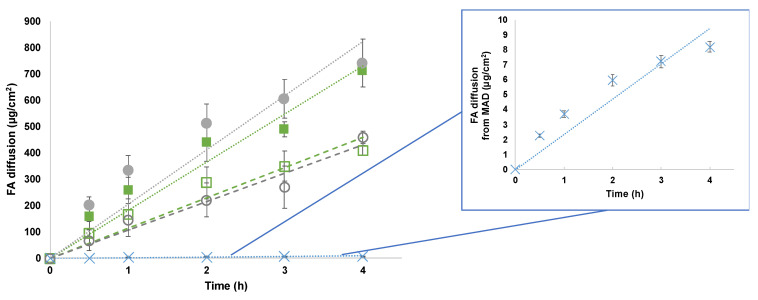
In vitro diffusion profiles of FA from TB1 (closed squares), TP1 (open squares), TB2 (closed circles), TP2 (open circles), and a MAD (crosses). The insert shows the enlargement of the in vitro diffusion profiles from the MAD. Data represent the mean of four independent experiments ± s.d.

As mentioned above, the specific composition of the produced LBNs affected their shape, dimension, encapsulation, and stability. In addition, a crucial point in their characterization is represented by the influence of the composition on the drug release. With this aim, Franz-cell experiments have been conducted to investigate FA diffusion through a synthetic membrane, namely a nylon membrane, in a physiological medium of PBS 1× [38]. UV was used to quantify FA within the receiving compartment and FA diffusion has been expressed as µg/cm^2^ against time (hours). The behavior of FA diffusion in TFs and a MAD are shown in Figure 3, considering the linear profile within 4 h.

Figure 3 shows that the difference in FA diffusion profiles depends on the nanosystem composition but seems to be independent of the particle size. Although there is evidence in the literature that a smaller nanocarrier particle size increases passive diffusivity [39], in this study, both TP1-FA and TB1-FA were characterized by a larger size compared with TP2-FA and TB2-FA (see Table 2), which showed a higher release. In addition, MAD-FA, characterized by a mean size close to that of TP2-FA and TB2-FA, showed a much slower release, which is possibly ascribed to the presence of different assembled nanostructures (i.e., lamellae and hexagonal and cubic phases). On the other hand, the dramatically lower FA diffusion from the MAD as compared to TFs suggests that the hydration medium plays a fundamental role in controlling FA release. Indeed, the presence of poloxamer 188 micelles within the aqueous dispersing phase in TP1-FA and TP2-FA could interfere with the drug accommodation within the micelle core or the vesicle bilayer, thus influencing its extrusion and diffusion, as reported in literature [24,40].

To better compare the nanosystems’ behavior, the diffusion coefficient values, expressed as fluxes, are reported in Table 4. Precisely, J_s_ values correspond to the slopes of the diffusion profiles, while J_n_ values are normalized fluxes as a function of the real FA concentration of each formulation. 

From the reported data, it is evident that the FA diffusion (J_n_) obtained from MAD was about 35-fold lower with respect to TB2-FA. On the other hand, the J_n_ values of the TB1-FA and TB2-FA systems demonstrated faster control in the diffusion of the drug compared with those of TP1-FA and TP2-FA at 2.88-fold and 1.71-fold, respectively. Hence, both the TFs composed of poloxamer 188 as the hydrating phase displayed good control in FA diffusion, confirming the potential influence of the mixed composition. 

From the analyses of mathematical data comparing the different releases, as summarized by Table 4, it is clearly evident that MAD-FA, TP1-FA, and TB2-FA follow Higuchi kinetics, while TB1-FA and TP2-FA follow first-order and zero-order kinetics, respectively.

Although a zero-order release may be highly desirable as it maintains the drug concentration in the environment at a constant level for extended periods, in this study, only the formulation containing T20 and hydrated with P satisfies the desired criteria. Perhaps the interaction between T20 and the poloxamer micelles allows for the formation of a network that slowly releases the drug. On the other hand, TB1-FA follows first-order kinetics, meaning that the rate of diffusion is proportional to the amount of drug remaining in the inner compartment, such that the amount of drug released over time decreases. This could be attributed to the incomplete dissolution of the drug (FA) in the nanosystem containing S20 and hydrated with Palitzsch buffer. Concerning the other formulations that follow Higuchi kinetics, namely TB2-FA, TP1-FA, and MAD-FA, in these cases, the nanosystems behave like a matrix system in which the initial drug concentration is much higher than the drug solubility, the drug particles are much smaller than the system thickness, and the drug diffusivity is constant [2,41].

### 3.4. In Vitro Antioxidant Activity 

The well-known activity of FA as a radical-scavenging agent in both the cosmetic and pharmaceutical fields [2,41] led us to evaluate the antioxidant activity of FA-loaded LBNs using two different assays with the aim of providing a wider profile of antioxidant activity in terms of both radical removal (DPPH) and ferric-ion reduction potential (FRAP). The obtained results, summarized in Table 5, demonstrated that the IC_50_ values and the FRAP antioxidant capacity of FA monitored one month from production were maintained either in solution or in an LBN without statistically significant changes. Finally, the slight decrease in both radical scavenging activity and antioxidant potential of FA when delivered by TB2 and TP2 allowed the selection of TB1 and TP1 as vesicular systems for ex-vivo experiments. 

### 3.5. In Vivo Patch Test Results

**Figure 4:**
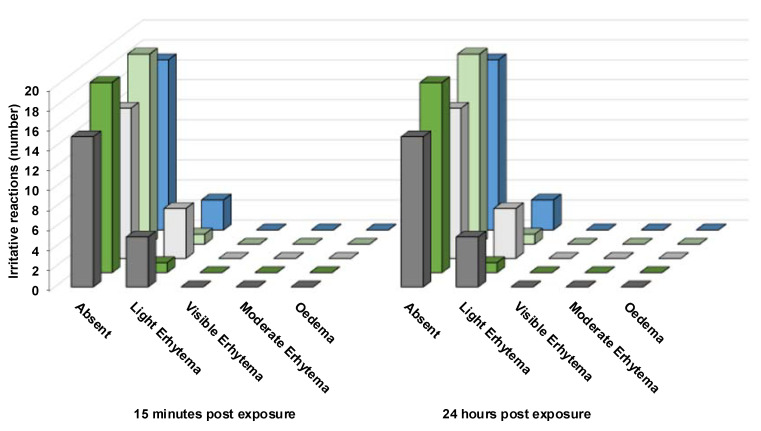
In vivo irritative reactions of TB1 (grey), TP1 (green), TB2 (white), TP2 (light green), and MAD (blue) on 20 healthy volunteers, acquired by patch test.

A patch test was performed to evaluate the potential irritation caused by the produced LBN formulations. Specifically, the formulations were applied onto the skin of 20 healthy volunteers, and the obtained results were expressed as a percentage of irritation reactions (Figure 4).

It was found that all the produced LBN formulations applied for 48 h under occlusive conditions are safe; therefore, these can be classified as non-irritant. Specifically, when comparing TFs, a slight difference can be observed for TB1-FA and TB2-FA, where the percentage of cases with no irritation decreased to 75%, while for TP1-FA and TP2-FA, 95% of cases showed no irritation and 5% of cases led to negligible reactions. In the case of MAD-FA, the percentage of absent reactions was 85%. 

These results allow us to propose these LBN formulations as suitable systems for topical application.

### 3.6. Effect of an FA-Loaded LBN on Skin Integrity and Morphology

**Figure 5:**
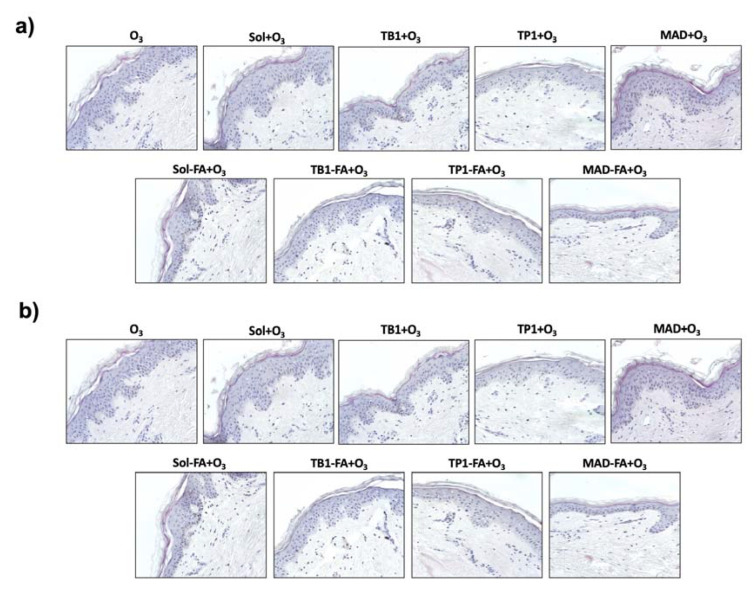
H/E staining of human skin biopsies pre-treated for 24 h with FA-loaded nanosystems exposed to 0.4 ppm of O_3_ for 4 h (**b**) or unexposed (**a**). Samples were collected 24 h after the end of the O_3_ exposure (T24). Pictures are representative of 1 donor.

To evaluate whether the FA-loaded LBN could affect the cutaneous tissue morphology or protect the skin against O_3_ exposure, hematoxylin/eosin staining was performed. As shown in Figure 5, the FA-unfilled LBN did not display any detrimental effect on human skin morphology, whereas O_3_ exposure could induce a visible damaging effect in the skin structure, especially in the epidermis. The treatment with FA-containing ethanol solution (Sol-FA) and TB1-FA displayed a slight irritating effect on the cutaneous tissue compared with control tissues, whereas TP1-FA and MAD-FA formulations did not affect the skin. These data suggest a possible irritating effect of the two formulations compared with the others, which was already visible in the basal condition. Of note, the treatment with TP1-FA and MAD-FA seemed to prevent the damaging effect of O_3_, while the unloaded formulations did not protect from the O_3_ detrimental damage, indicating that FA exerts a protective role only when delivered by TP1 and MAD. Indeed, Sol-FA and TB1-FA still displayed an irritating effect on O_3_-exposed human skin compared with the other formulations.

### 3.7. Protective Effect of FA-Loaded LBNs against the O_3_-Induced Cutaneous Oxidative Damage 

**Figure 6:**
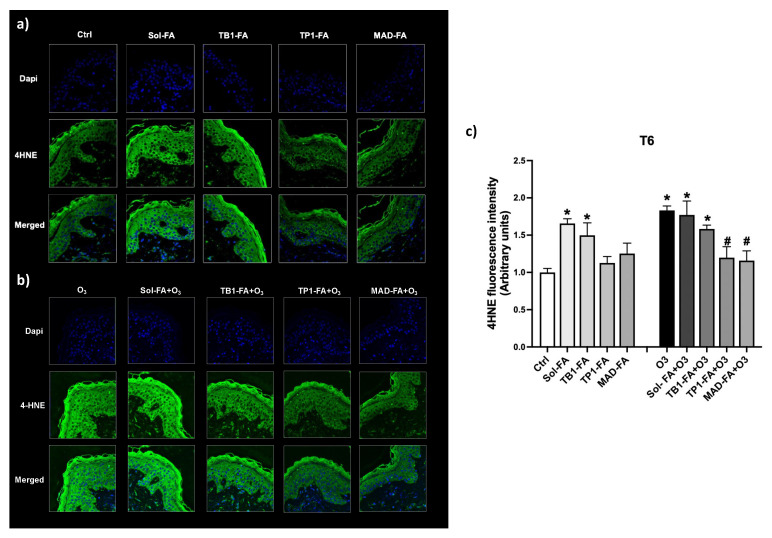
(**a**,**b**) Levels of 4HNE in human skin biopsies exposed to 0.4 ppm of O_3_ for 4 h, pre-treated with formulations loaded with FA for 24 h, and then collected 6 h post-exposure (T6). Green staining represents 4HNE; the blue staining (DAPI) represents nuclei. Pictures were taken at 40× magnification. (**c**) Graphical representation of the 4HNE fluorescent signal quantified by using ImageJ software. Data are expressed as the mean of three different experiments ± s.d.; * *p* < 0.05 for formulations +/− O_3_ vs. Ctrl and # *p* < 0.05 for formulations + O_3_ vs. O_3_ by 2-way ANOVA followed by Tukey’s post hoc comparison test.

Ozone (O_3_) is one of the most recognized oxidative air pollutants that can react with skin macromolecules such as lipids, proteins, etc. and induce oxidative stress reactions culminating in the formation of lipid peroxidation products like the reactive aldehyde 4-hydroxy-nonenal (4HNE) [42]. 4HNE can then promote the formation of protein adducts within the skin that can alter the cutaneous homeostasis, favoring an oxidative environment. The antioxidant property of the different formulations loaded with FA was evaluated in skin tissues exposed to O_3_ by assessing the immunofluorescence staining for 4HNE adduct levels. As depicted in Figure 6, TP1-FA and MAD-FA could prevent O_3_-induced oxidative damage by reducing the levels of 4HNE to control levels, whereas Sol-FA and TB1-FA did not display any protective effect. Of note, skin explants treated with Sol-FA and TB1-FA already displayed increased levels of 4HNE in the basal condition, confirming the previous data on skin morphology and suggesting that these formulations can be irritating for human skin. These data suggest that the TB1 nanosystem may not be able to properly deliver FA throughout the skin, which in turn accumulates, causing cutaneous irritation, whereas TP1 and MAD may represent good candidates for FA cutaneous permeation, favoring the protection against O_3_-induced oxidative damage. 

## 4. Conclusions

This study compares two distinct lipid-based nanocarriers, namely transferosomes and MADs, as delivery systems for the topical cutaneous application of the natural antioxidant ferulic acid. It was discovered that these systems are able to efficiently encapsulate the drug while preserving its antioxidant properties.

Additionally, the Franz-cell experiment outcomes revealed that the engineered LBN, which incorporates poloxamer 188 in its composition, generates a structure similar to Chinese boxes, capable of controlling the drug release. Indeed, poloxamer 188 micelles dispersed in the hydrating aqueous phase can retain FA, resulting in a slower drug diffusion through the diffusion membrane. It should be noted, however, that the type of non-ionic surfactant used can influence the release of the drug.

Regarding FA diffusion from the MAD, the profile displayed a higher rate of diffusion compared with TFs. From an in vivo perspective, patch tests demonstrated that all the manufactured LBNs, when applied for 48 h under occlusive conditions, were deemed safe.

The final evaluation aimed to demonstrate whether FA-containing formulations could alter cutaneous tissue morphology or offer skin protection against ozone (O_3_) exposure. These assessments, performed using hematoxylin/eosin and 4HNE staining, suggested that treatment with TP1-FA and MAD-FA formulations could counteract the damaging effects of O_3_, whereas the unloaded formulations failed to provide any protection from O_3_’s harmful impacts. Considering that O_3_ is able to react with skin macromolecules such as lipids, proteins, etc., inducing oxidative stress reactions and culminating in the formation of ROS and lipid peroxidation products, we suggest that FA, when loaded into TP1 and MAD, can directly scavenge O_3_− reactive species, preventing the development of an oxidative environment. Indeed, as summarized by Zduńska and collegues [2], FA can prevent the formation of reactive oxygen species (ROS) by inhibiting enzymes that catalyze the generation of free radicals or by enhancing scavenger enzyme activity. FA antioxidant activity is primarily due to its structure that is enriched in hydroxyl and methoxy groups attached to the phenyl ring, which enables it to bind transition metals such as iron and copper that involved in ROS production and lipid peroxidation events. Thus FA, directly interacting with the radical molecule, can form stable phenoxyl radicals. In addition, FA can donate hydrogen to radicals to stabilize them, thus preventing membrane lipids from undergoing oxidative processes and the development of lipid peroxidation products such as reactive aldehydes [2].

In conclusion, these findings provide a promising platform for the use of LBNs such as TFs in the topical application of natural antioxidant molecules.

## Figures and Tables

**Table 1:** Composition of the prepared LBNs.

Acronym	Composition					
	Molar Ratio				Aqueous Phase (Type)	Lipid Phase (% of Total Weight)
	*PC*	*CH*	*S20*	*T20*		
TB1	4	2	1	-	Palitzsch buffer	2.5
TP1	4	2	1	-	Poloxamer 188 (2.5% *w*/*w*)	2.5
TB2	4	2	*-*	1	Palitzsch buffer	2.5
TP2	4	2	*-*	1	Poloxamer 188 (2.5% *w*/*w*)	2.5
MAD	Weight (g)					
	*GMO*		*Pluronic 188*		*water*	5
	2.25		0.25		47.5	

**Table 2:** Size and polydispersity of FA-loaded LBNs over time, as determined by PCS.

Time (d)	TB1-FA Z-Av (nm) *PdI*	TP1-FA Z-Av (nm) *PdI*	TB2-FA Z-Av (nm) *PdI*	TP2-FA Z-Av (nm) *PdI*	MAD-FA Z-Av (nm) *PdI*
1	442.9 ± 32.1	412.2 ± 27.6	269.2 ± 15.4	207.3 ± 16.3	283.0 ± 12.9
	*0.417 ± 0.023*	*0.304 ± 0.018*	*0.276 ± 0.004*	*0.249 ± 0.010*	*0.113 ± 0.029*
7	449.2 ± 25.4	514.1 ± 35.7	337.1 ± 21.8	230.2 ± 21.5	265.4 ± 14.2
	*0.442 ± 0.025*	*0.260 ± 0.023*	*0.296 ± 0.013*	*0.283 ± 0.012*	*0.122 ± 0.034*
15	454.6 ± 30.7	570.1 ± 48.3	269.0 ± 23.5	222.8 ± 16.1	261.6 ± 13.4
	*0.310 ± 0.009*	*0.170 ± 0.008*	*0.297 ± 0.025*	*0.286 ± 0.009*	*0.136 ± 0.002*
30	436.0 ± 26.8	515.8 ± 30.9	371.2 ± 19.6	231.4 ± 18.6	265.9 ± 9.8
	*0.428 ± 0.021*	*0.301 ± 0.011*	*0.312 ± 0.022*	*0.295 ± 0.007*	*0.103 ± 0.032*

**Table 3:** Shelf-life values of the LBNs produced.

Parameter	TB1-FA	TP1-FA	TB2-FA	TP2-FA	MAD-FA
K	0.0007	0.0034	0.0027	0.0031	0.0035
t_90_ (days) ^a^	150	31	39	34	30
t_1/2_ (days) ^b^	990	204	257	224	198

^a^ Time at which the drug concentration had decreased by 10%. ^b^ Time at which the drug concentration had decreased by 50%.

**Table 4:** Diffusion parameters of FA.

Acronym	Flux (μg/cm^2^ · h)		R^2^		
	J_s_	J_n_	Zero Order	First Order	Higuchi
TB1-FA	182.91	94.70	0.9644	0.9710	0.9576
TB2-FA	205.93	111.97	0.9447	0.9702	0.9921
TP1-FA	115.01	62.88	0.9556	0.9672	0.9825
TP2-FA	108.00	65.54	0.9632	0.9558	0.8939
MAD-FA	2.35	3.21	0.9336	0.9339	0.9916

**Table 5:** Antioxidant activity of FA-containing solutions and LBNs as determined by DPPH and FRAP assays.

Acronym	DPPH—IC_50_ (μg/mL)		FRAP—μmol TE/g	
	1 Day	30 Days	1 Day	30 Days
Sol-FA	12.33 ± 0.19	12.57 ± 0.02	5751.44 ± 232.68	5656.90 ± 330.04
TB1-FA	12.20 ± 0.04	12.34 ± 0.01	6023.32 ± 61.95	5915.40 ± 255.15
TP1-FA	13.71 ± 0.32	13.59 ± 0.06	5054.89 ± 447.45	5221.38 ± 160.06
TB2-FA	13.89 ± 0.18	13.55 ± 0.10	3637.15 ± 148.63	3890.23 ± 18.57
TP2-FA	15.97 ± 0.64	15.67 ± 1.10	3747.12 ± 4.08	4072.74 ± 356.38
MAD-FA	16.66 ± 0.14	17.79 ± 0.38	3036.95 ± 203.22	2937.63 ± 235.25

The values are the mean of three different experiments ± SEM.

## Data Availability

The data presented in this study are available from the corresponding author upon request. The data are not publicly available due to privacy restrictions.
